# New records of an alien aphid species Tinocallis (Sappocallis) takachihoensis from countries in central and northern Europe (Hemiptera, Aphididae, Calaphidinae)

**DOI:** 10.3897/zookeys.730.21599

**Published:** 2018-01-17

**Authors:** Mariusz Kanturski, Yerim Lee, Łukasz Depa

**Affiliations:** 1 Department of Zoology, Faculty of Biology and Environmental Protection, University of Silesia in Katowice, Bankowa 9, 40–007 Katowice, Poland; 2 Laboratory of Insect Biosystematics, Department of Agricultural Biotechnology, Research Institute of Agriculture and Life Sciences, Seoul National University, Seoul, Republic of Korea

**Keywords:** Aphidoidea, distribution, new record, Sternorrhyncha, *Ulmus glabra* ‘Pendula’

## Abstract

The aphid genus *Tinocallis* Matsumura, 1919 (Hemiptera: Aphididae: Calaphidinae) in central and northern Europe is reviewed; it includes four species. The first records of the Asian elm aphid *Tinocallis takachihoensis* Higuchi, 1972 are reported from the Czech Republic, Denmark, and Poland. The record in Denmark is the first in Scandinavia. Alate viviparous females and nymphs of *T. takachihoensis* were collected from *Ulmus glabra* “Pendula” (the Czech Republic and Poland) and from *U. minor* (Denmark) in 2017. *Tinocallis takachihoensis* is the fourth *Tinocallis* species to be recorded from Poland and together with two other species from the subgenus Sappocallis, *T. nevskyi* and *T. saltans*, is a species of alien origin. The alate viviparous females are reviewed and re-described and their affinities and distribution in central and northern Europe are discussed. A key to the European species of *Tinocallis* based on the characters of live and slide-mounted specimens is also given.

## Introduction

Of the approximately 1590 aphid species described or recorded from Europe (Nieto Nafría et al. 2013) to date, approximately 103 species are known to be of an alien origin from North America and Asia (Coeur d’acier et al. 2010, Pérez Hidalgo et al. 2011; Rakauskas 2011; Panini et al. 2017). The area of Poland is one of the best recognised in terms of aphid fauna in Europe with 766 recorded species and subspecies (Wojciechowski et al. 2015; Kanturski et al. 2017) to date. In Poland, as many as 34 species of an alien origin were reported earlier (Wieczorek 2011). However, this number is continuously changing due to ongoing research and biological invasions (Kanturski et al. 2017; Walczak et al. 2017).

The aphid genus *Tinocallis* Matsumura, 1919 (Calaphidinae) comprises 18–19 species within four subgenera (Favret 2017; Lee and Lee 2017) and is the second largest *Panaphidini* genus (Quednau 2001; Favret 2017). The species of this genus are characterised by a small and delicate body and are mostly associated with Ulmaceae (Blackman and Eastop 1994, 2017). All of the viviparae are alate and they usually have paired spinal and marginal tubercular processes on the thorax and abdomen. The secondary rhinaria on antennal segment III are narrow, transversely elongated, or slit-like. The genus has caused many taxonomical difficulties due to its seasonal variations (Quednau 2001).

The Asian elm aphid, *Tinocallis takachihoensis* Higuchi, 1972, is one of the members of the subgenus Sappocallis Matsumura, 1919. This species was originally described from Japan and is known to be primarily distributed in Eastern Asia (Higuchi 1972). *Tinocallis takachihoensis* along with *T. saltans* (Nevsky, 1929), *T. ulmiparvifoliae* Matsumura, 1919, and *T. zelkowae* (Takahashi, 1919) have been introduced to other parts of the world (Blackman and Eastop 2017; Foottit et al. 2006; Quednau and Shaposhnikov 1988). In Europe, this species was reported for the first time from France (from a suction trap) (Leclant 1986). Since then, this species has been reported from the United Kingdom (Döring 2007, 2008) and the Mediterranean area (Patti and Barbagallo 1997; Mier Durante and Pérez Hidalgo 2002; Barbagallo and Massimino Cocuzza 2014). Although recent observations of the occurrence of this species come from Greece (Papapanagiotou et al. 2012), Germany, and the Netherlands (Piron 2013), Piron’s information comes from a personal communication from Schrameyer who received the name of the species in question from Thieme (T. Thieme, pers. comm.).

In this paper, the occurrence of *T. takachihoensis* from the Czech Republic, Denmark, and Poland is reported for the first time. Its affinities to other known *Tinocallis* species from these regions are also discussed and a morphological key to the identification of all European representatives of this genus is provided.

## Material and methods

Alate viviparous females and nymphs of *Tinocallis takachihoensis* were collected in Opava (the Czech Republic) on 19.08.2017, Katowice (Poland) on 12.07.2017, and Copenhagen (Denmark) on 16.06.2017. The aphids were transferred to 80% ethanol and prepared after the Kanturski and Wieczorek (2012) protocol. In-life photographs were taken by the Sony SLT digital camera with the Sigma EX 50 mm lens with intermediate rings. Mounted specimens were examined by a Nikon Eclipse E600 and photographed by Nikon DS-Fi camera. The slides are deposited in the Aphidoidea collection of the Department of Zoology, University of Silesia in Katowice, Poland (UŚ).

The following abbreviations are used:

**BL** body length;

**HW** greatest head width across compound eyes;

**ANT** antennae or their lengths;

**ANT I, II, III, IV, V, VI** antennal segments I, II, III, IV, V, VI or their lengths (ratios between antennal segments are simply given as e.g. ‘VI: III’);

**BASE** basal part of last antennal segment or its length;

**PT** processus terminalis of last antennal segment or its length;

**URS** ultimate segments of rostrum (IV + V) or their length;

**Cu_1a_** first cubital vein;

**Cu_1b_** second cubital vein;

**FEMORA III** hind femora length;

**TIBIAE III** hind tibiae length;

**HT I** first segment of hind tarsus,

**HT II** second segment of hind tarsus or its length;

**SIPH** siphunculi or their length.

For morphological comparison slides of *Tinocallis* specimens from other localities were used. The studied material is deposited in the Natural History Museum in London, UK (**BMNH**), and Zoological Institute, Polish Academy of Sciences, Warsaw, Poland (**ZMPA**).

## Taxonomy

### Genus Tinocallis

Taxon classificationAnimaliaHemipteraAphididae

Matsumura, 1919

Figs 1 , 2 , 3 , 4 , 5 , 6 , 7 , 8

Tinocallis Matsumura, 1919: 100.
Lutaphis Shinji, 1924: 346.

#### Diagnosis.

This genus can be recognized by having narrow transversely elongated or slit-like secondary rhinaria on ANT III, and ABD III, V, and VII with laterally displaced spinal dorsal setae. Abdominal tergites I and II usually have finger-like dorsal tubercles.

#### Type species.

*Tinocallis ulmiparvifoliae* Matsumura, 1919.

### Tinocallis (Sappocallis) takachihoensis

Taxon classificationAnimaliaHemipteraAphididae

Higuchi, 1972

Figs 1 , 2 , 3 , 4 , 5 , 6 , 7 , 8 ; Table 1

#### Redescription.

Alate viviparous female

**Figure 1.**
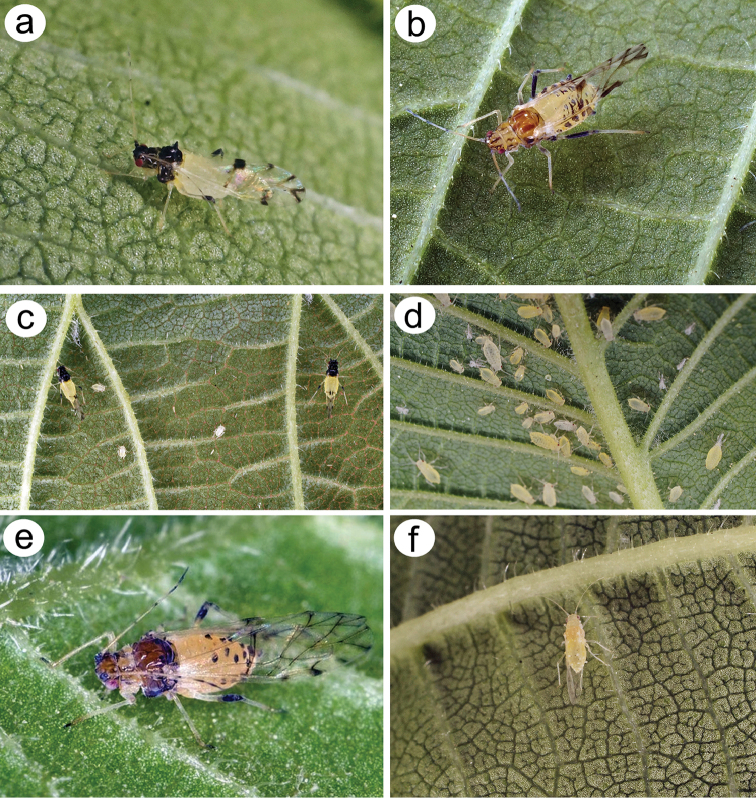
Live specimens of **a** *Tinocallis takachihoensis* **b** *T. platani* **c** *T. takachihoensis* with first instar larvae **d** larvae and nymphs of *T. platani* **e** *T. saltans* **f** *T. nevskyi*.

**Figure 2.**
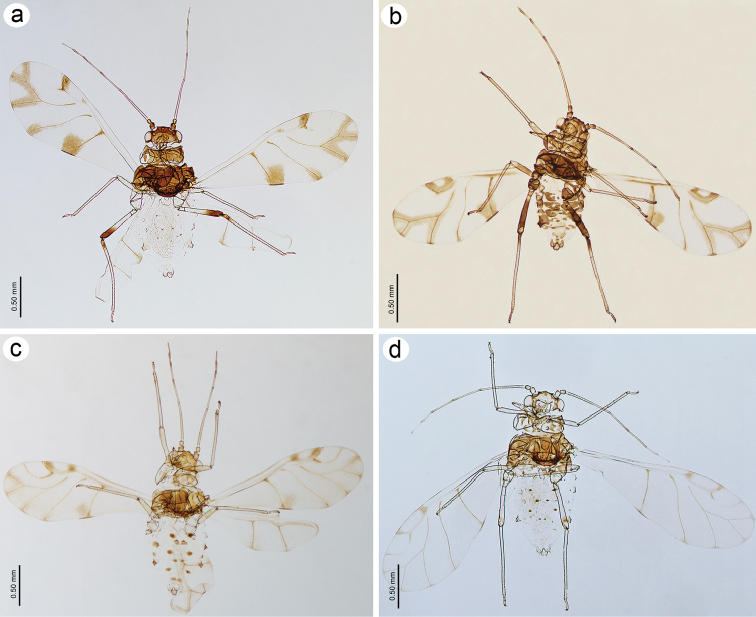
Mounted specimens of alate viviparous females of **a** *Tinocallis takachihoensis* **b** *T. platani* **c** *T. saltans* **d** *T. nevskyi*.

**Figure 3.**
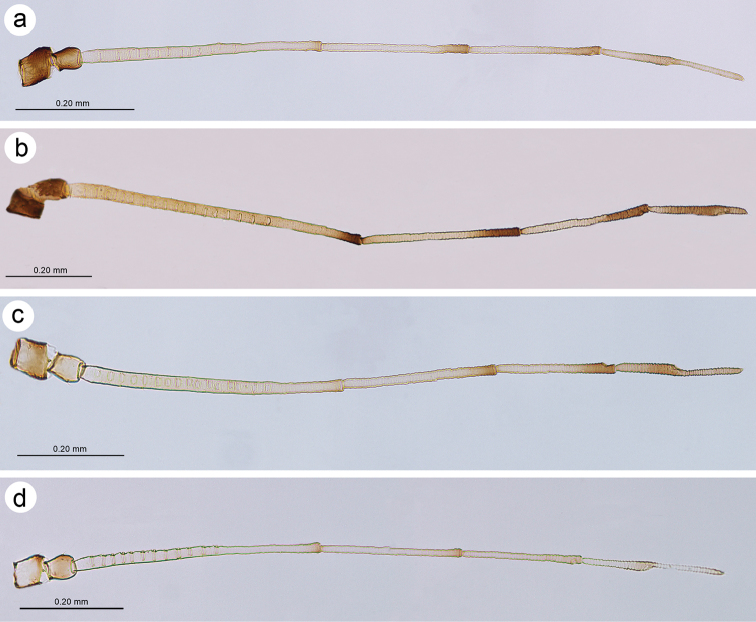
Antennae of alate viviparous females of **a** *Tinocallis takachihoensis* **b** *T. platani* **c** *T. saltans* **d** *T. nevskyi*.

**Figure 4.**
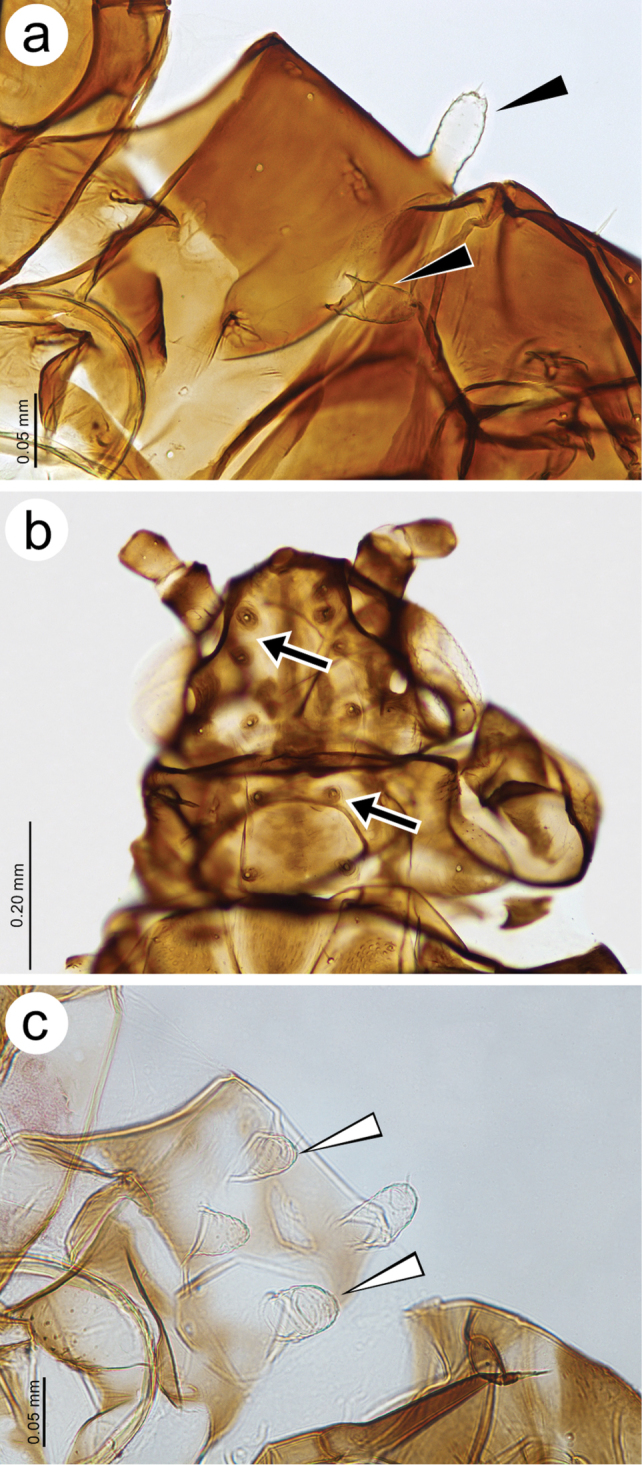
Head and pronotum features **a** pronotum of *Tinocallis takachihoensis* with one pair of projections (black arrowheads) **b** head and pronotum of *T. platani* without projections (arrows) **c** pronotum of *T. nevskyi* with two pairs of projections.

**Figure 5.**
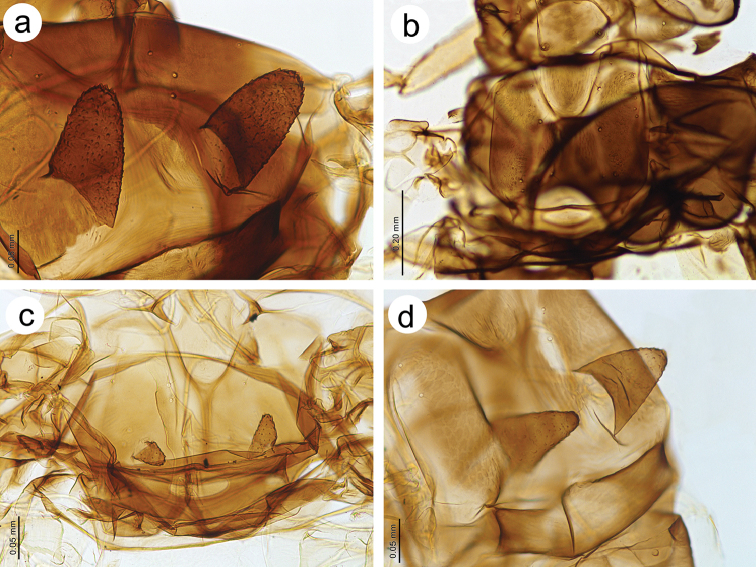
Mesonotum of **a** *Tinocallis takachihoensis* **b** *T. platani* (without projections) **c** *T. saltans* **d** *T. nevskyi*.

**Figure 6.**
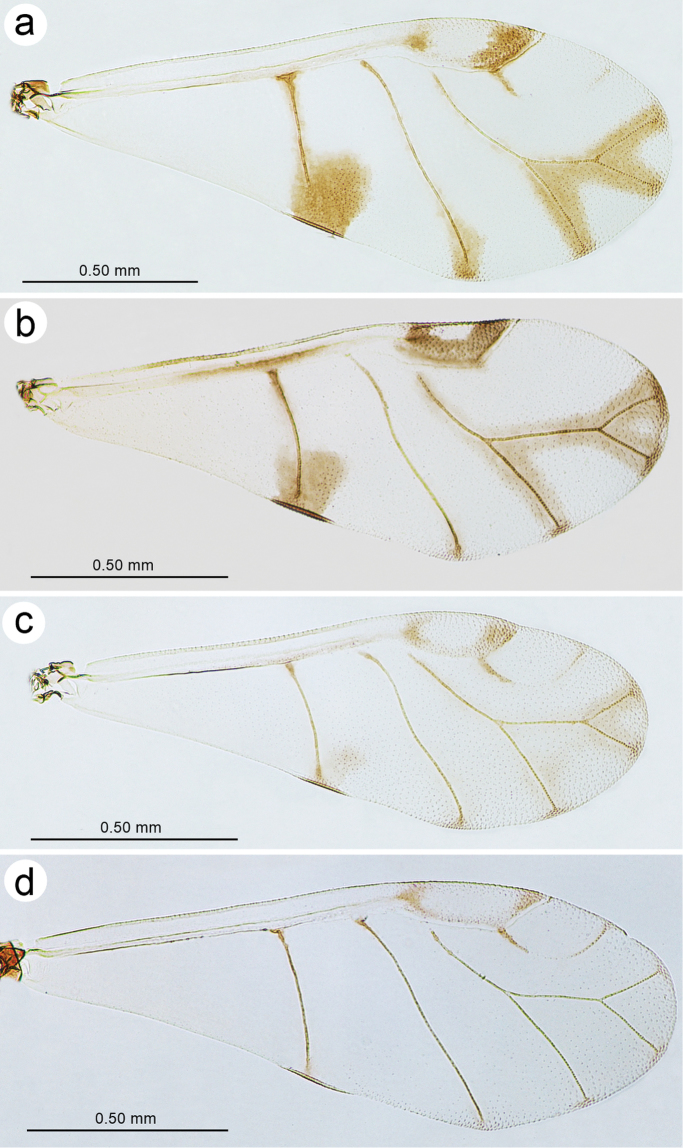
Forewing pigmentation of **a** *Tinocallis takachihoensis* **b** *T. platani* **c** *T. saltans* **d** *T. nevskyi*.

**Figure 7.**
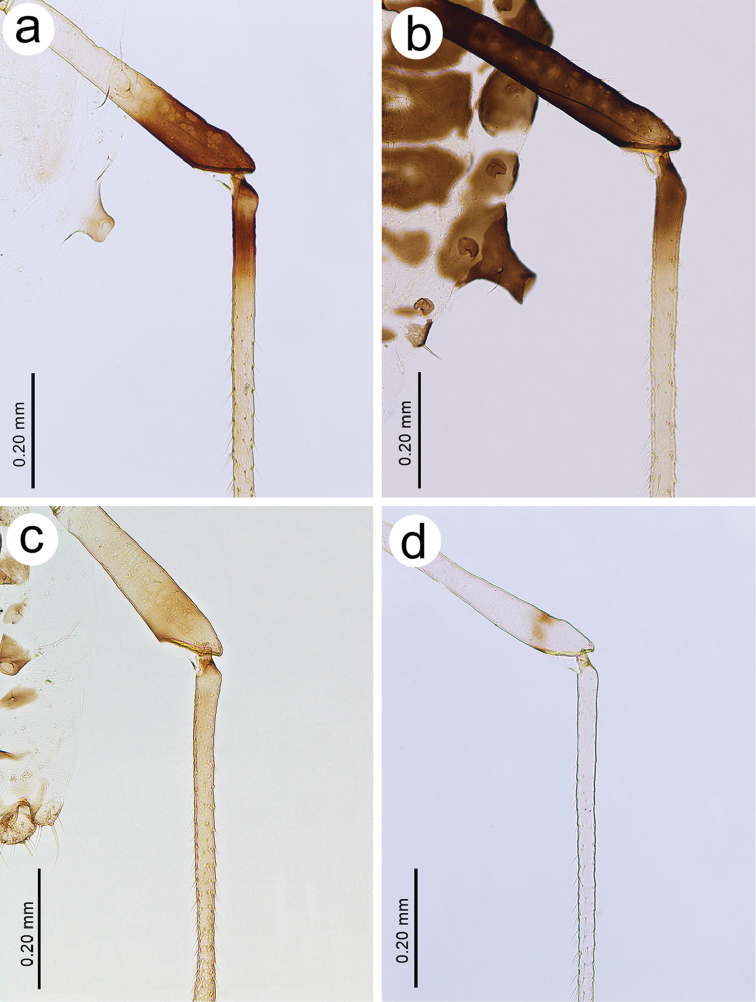
Hind leg pigmentation of **a** *Tinocallis takachihoensis* **b** *T. platani* **c** *T. saltans* **d** *T. nevskyi*.

**Figure 8.**
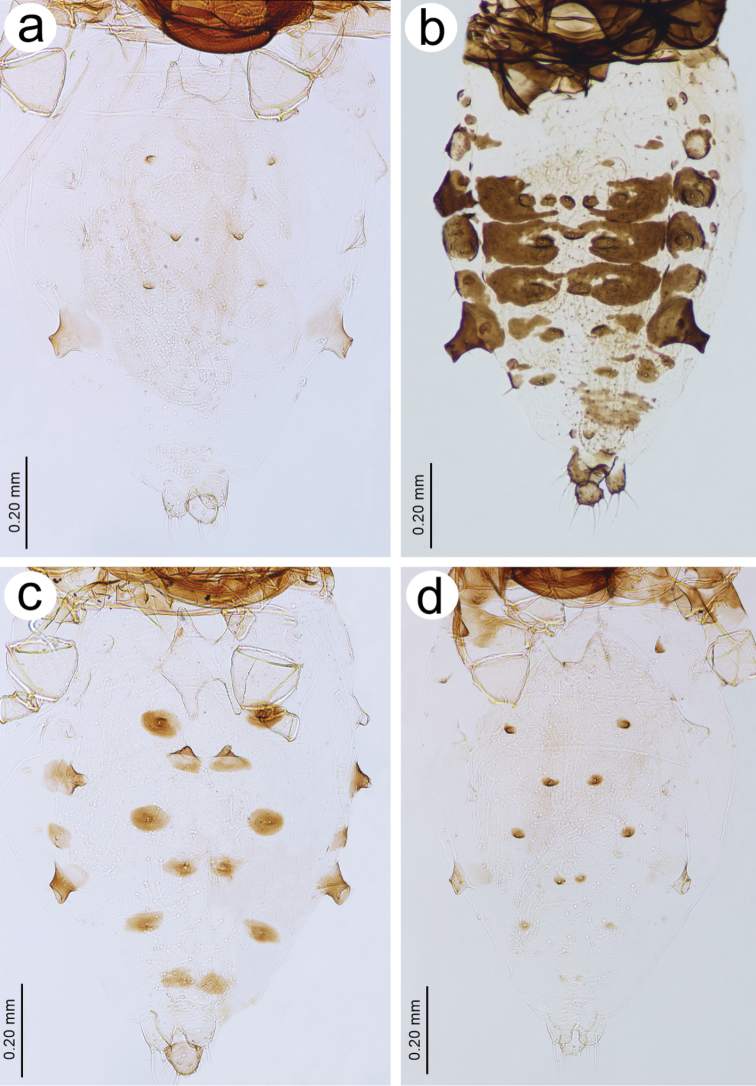
Abdominal sclerotization of Central and North European *Tinocallis*: **a** *T. takachihoensis* **b** *T. platani* **c** *T. saltans* **d** *T. nevskyi*.

*Colour*. Colour of live specimens: head and thorax black, ANT pale yellow with dark apices of segments, fore and middle legs pale yellow, hind legs pale yellow with black distal part of femora and proximal part of tibiae. Wings pigmented on the area of pterostigma, media and Cu_1b_. Abdomen yellow (Fig. 1a, c). Pigmentation of mounted specimens: head and thorax brown (Fig. 1a). ANT pale with brown ANT I and ANT II and slightly darker apices of flagellar segments (Fig. 2a). *Morphometric characters*. ANT 0.76–1.00 × BL and 0.26–0.27 × HW. ANT III with 17–21 slit-like secondary rhinaria (Fig. 3a). PT 1.13–1.19 × BASE. Other antennal ratios: ANT VI/ANT III 0.57–0.64, ANT V/ANT III 0.52–0.54, ANT IV/ANT III 0.52–0.61. Ant I with 3 setae, Ant II with 2–3, ANT III with 9–13 setae, ANT IV with 2 setae, ANT V with 2–3 setae, ANT VI with one basal seta. URS 0.18 × ANT III, 0.28–0.32 × ANT VI, and 0.90–1.00 × HT II. Pronotum with two finger-like projections on the distal part (Fig. 4a). Mesonotum with two large, imbricated projections with rounded apices (Fig. 5a). HT II 0.18–0.20 × ANT III and 0.31–0.32 × ANT VI. Forewings with distal branches of media bordered with fuscous and with more-or-less extensive fuscous patches at distal ends of Cu and Cu_1b_ (Fig. 6a). Hind legs with dark distal part of femora and proximal part of tibiae (Fig. 7a). Abdomen pale, SIPH pale on basal part and pale brown on apex. Abdomen without sclerotisation besides very small darker projections on ABD III–V (Fig. 8a).

**Table 1.** Measurements (in mm) of alate viviparous females of *Tinocallis* species studied.

Character	*T. takachihoensis* n = 20	*T. platani* n = 13	*T. saltans* n = 9	*T. nevskyi* n = 15
BL	1.27–1.47	1.75–2.25	1.50–1.75	1.72–1.90
HW	0.39–0.46	0.45–0.46	0.40–0.45	0.40–0.42
ANT	1.48–1.73	1.65–1.77	1.06–1.35	1.34–1.48
ANT III	0.50–0.59	0.60–0.70	0.38–0.49	0.45–0.47
ANT IV	0.26–0.36	0.36–0.39	0.21–2.28	0.27–0.30
ANT V	0.27–0.31	0.31–0.32	0.17–0.22	0.22–0.28
ANT VI	0.32–0.34	0.21–0.22	0.19–0.23	0.29–0.32
BASE	0.15	0.17–0.18	0.10–0.12	0.14
PT	0.17–0.18	0.04	0.09–0.11	0.15–0.18
URS	0.09–0.11	0.12–0.13	0.05–0.06	0.085
III FEMUR	0.38–0.58	0.48–0.58	0.36–0.40	0.43–0.45
III TIBIA	0.74–1.06	1.00–1.20	0.63–0.70	0.74–0.79
HT I	0.03–0.04	0.04–0.05	0.03	0.02–0.03
HT II	0.10–0.11	0.11–0.12	0.10	0.09
Forewings	1.82–2.50	2.25–2.75	1.75–2.00	2.05–2.25
Hind wings	1.00–1.80	1.37–1.75	1.20–1.55	1.25–1.37
SIPH	0.04–0.05	0.07–0.10	0.05–0.06	0.03–0.04
cauda length	0.05–0.06	0.07–0.08	0.07	0.05
cauda width	0.05–0.06	0.06–0.08	0.05–0.07	0.06
genital plate length	0.07–0.08	0.09–0.10	0.08–0.09	0.07–0.08
genital plate width	0.12–0.15	0.17–0.20	0.11–0.12	0.13–0.18

#### Material examined.

Six alate viviparous females, **CZECH REPUBLIC**: Moravskoslezský kraj (Opava and vicinity), 18 Aug 2017, No. 08/17/5, on *Ulmus glabra*, coll. M. Kanturski (UŚ); five alate viviparous females, **DENMARK**: North East Zealand (Copenhagen), 18 Jun 2017, No. 05/17/3, on *Ulmus* sp., coll. M. Kanturski (UŚ); 15 alate viviparous females, **POLAND**: Upper Silesia (Katowice), 12 Jul 2017, No. 07/172b, on *Ulmus glabra* ‘Pendula’, coll. M. Kanturski (UŚ).

#### Additional material examined.

six alate viviparous females, CHINA: Xiangshan Botanic Garden, 25 May1985, No. VFE18142, RLB3615, on *Ulmus* sp., coll. R. Blackmam & V. Eastop (BMNH); three alate viviparous females, UNITED KINGDOM: Humberside, 16 Oct 1997, No. BM1999–7 on *Zelkova serrata*, coll. not known (BMNH).

### Tinocallis (Eotinocallis) platani

Taxon classificationAnimaliaHemipteraAphididae

(Kaltenbach, 1843)

Figs 1 , 2 , 3 , 4 , 5 , 6 , 7 , 8 ; Table 1

#### Redescription.

Alate viviparous female

*Colour*. Colour of live specimens: head and prothorax yellow with brown longitudinal stripes. The rest of thorax brown. ANT pale yellow with dark apices of segments, fore and middle legs pale yellow, hind legs with black femora and proximal part of tibiae. Wings pigmented on the area of pterostigma, media and Cu_1b_. Abdomen yellow with brown extensive spots on the dorsal side (Fig. 1b). Nymphs pale yellow (Fig. 1d). Pigmentation of mounted specimens: head and thorax brown. ANT pale with brown ANT I, ANT II and slightly darker apices of flagellar segments (Fig. 2b).

*Morphometric characters*. ANT 0.78–0.94 × BL and 0.25–0.27 × HW. ANT III with 16–26 slit-like secondary rhinaria (Fig. 3b). PT 0.23–0.25 × BASE. Other antennal ratios: ANT VI/ANT III 0.31–0.35, ANT V/ANT III 0.45–0.51, ANT IV/ANT III 0.55–0.60. Ant I with 3–4 setae, Ant II with 4, ANT III with 10–15 setae, ANT IV with 3–5 setae, ANT V with 2–4 setae, ANT VI with one basal seta. URS 0.18–0.20 × ANT III, 0.57 × ANT VI, and 1.04–1.09 × HT II. Pronotum without projections. Only very small protuberances, similar to those on the head are visible (Fig. 4b). Mesonotum without projections (Fig. 5b). HT II 0.17–0.18 × ANT III and 0.52–0.55 × ANT VI. Forewings without a radial sector. Stigma and media strongly bordered with fuscous basally as well as distally, with a dark spot over Cu_1b_ (Fig. 6b). Hind legs with uniformly dark brown femora and proximal part of tibiae (Fig. 7b). Abdomen robust, pale with brown more or less extensive dorsal sclerites or broken crossbars. SIPH dark brown (Fig. 8b).

#### Material examined.

three alate viviparous females, GERMANY: Lehmen, 18 Aug 1936, No. BM1984–340, on *Ulmus effusa* (= *U. laevis*), coll. D. Hille Ris Lambers (BMNH); three alate viviparous females, POLAND: Piekary Śląskie, 19 May 2015, No. 04/15/33 on *Ulmus* sp., coll. M. Kanturski & Ł. Depa (UŚ); three alate viviparous females, Warszawa, 13 May 1962, No. R3953, on *Ulmus laevis*, coll. H. Szelegiewicz (ZMPA); four alate viviparous females, UNITED KINGDOM: London, Kew, 29 Jun 1965, No. BM1982–492, on *Ulmus laevis*, coll. H.L.G Stroyan (BMNH).

### Tinocallis (Sappocallis) saltans

Taxon classificationAnimaliaHemipteraAphididae

(Nevsky, 1929)

Figs 1 , 2 , 3 ; 5 , 6 , 7 , 8 ; Table 1

#### Redescription.

Alate viviparous female.

*Colour.* Colour of live specimens: head brown, ANT yellow with distinctly darker apices of ANT IV and V and almost whole ANT VI. Prothorax yellow or light brown, the rest of thorax dark brown to dark brown. Legs pale with visible darker distal part of hind femora. Abdomen yellow with brown with brown, small and rounded sclerites (Fig. 1e). Pigmentation of mounted specimens: head and thorax light brown to brown with lighter prothorax. The rest of body pale (Fig. 2c).

*Morphometric characters*. ANT 0.71–0.77 × BL and 0.33–0.37 × HW. ANT III with 12–17 transverse oval secondary rhinaria (Fig. 3c). PT 0.95 × BASE. Other antennal ratios: ANT VI/ANT III 0.47–0.51, ANT V/ANT III 0.44, ANT IV/ANT III 0.55–0.57. Ant I with 3 setae, Ant II with 2, ANT III with 10–11 setae, ANT IV with 2–4 setae, ANT V with 2 setae, ANT VI with one basal seta. URS 0.10–0.15 × ANT III, 0.21–0.30 × ANT VI, and 0.50–0.60 × HT II. Pronotum with four projections, two on distal and two on proximal part (Fig. 4c). Mesonotum with two small, imbricated projections on the distal part with rounded apices (Fig. 5c). HT II 0.20–0.26 × ANT III and 0.42–0.51 × ANT VI. Forewings with base of pterostigma with dark spot and pigmentation or bordering on branches of media and delicate light brown spot near Cu_1b_ (Fig. 6c). Hind legs pale or light brown with darker distal part of femora (Fig. 7c). Abdomen pale with brown sclerites with distinct tubercles on ABD III-V. SIPH brown (Fig. 8c).

#### Material examined.

three alate viviparous females, CHINA: Xiangshan, 25 May 1985, No. VFE 18128, on *Ulmus* sp., coll. V.F. Eastop (BMNH); two alate viviparous females POLAND: Katowice, 17 Jul 2016, No. 07/16/10, on *Ulmus* sp., coll. M. Kanturski leg., UŚ; two alate viviparous females, TAJIKISTAN: Gissarskij cgrebt (1300m), 03 Jul 1959, No. R.3964, on *Ulmus campestris*, coll. M. Narzikulov (ZMPA); two alate viviparous females, UKRAINE: Kanevskij zapov., 17 Aug 1945, No. R.3961, on *Ulmus* sp., coll. V. Mamontova (ZMPA).

### Tinocallis (Sappocallis) nevskyi

Taxon classificationAnimaliaHemipteraAphididae

Remaudière, Quednau & Heie, 1988

Figs 1 , 2 , 3 , 4 , 5 , 6 , 7 , 8 ; Table 1

#### Redescription.

Alate viviparous female

*Colour.* Colour of live specimens: whole body pale yellow to whitish yellow with poorly visible very small, brown abdominal sclerites. Wings not pigmented (Fig. 1f). Pigmentation of mounted specimens: head pale with light brown edges, ANT pale, pronotum pale with light brown longitudinal stripes. The rest of thorax brown (Fig. 2d).

*Morphometric characters*. ANT 0.78 × BL and 0.28–0.29 × HW. ANT III with 13–15 slit-like secondary rhinaria (Fig. 3d). PT 1.07–1.24 × BASE. Other antennal ratios: ANT VI/ANT III 0.64–0.69, ANT V/ANT III 0.50–0.59, ANT IV/ANT III 0.60–0.63. ANT I with 2–3 setae, ANT II with 2, ANT III with 7–10 setae, ANT IV with 2–3 setae, ANT V with 2–3 setae, ANT VI with one basal seta. URS 0.18 × ANT III, 0.26–0.29 × ANT VI, and 0.09–0.94 × HT II. Pronotum with four projections, two on distal and two on proximal part (Fig. 4c). Mesonotum with two large and wide basally, imbricated projections on the distal part (Fig. 5d). HT II 0.20 × ANT III and 0.29–0.31 × ANT VI. Forewings with apices of media and Cu with very small, poorly visible spots (Fig. 6d). Hind legs pale with small dark spot on distal part of femora, but the end of femora also pale (Fig. 7d). Abdomen, pale with brown, very small dorsal sclerites on ABD III-VIII but those on ABD VII and VIII poorly visible. SIPH pale brown (Fig. 8d).

#### Material examined.

three alate viviparous females, AFGHANISTAN: Kabul, 08 May 1975, No. BM 1984–340, on *Ulmus* sp., coll. R. van den Bosch (BMNH); four alate viviparous females, POLAND: Katowice (first record for Upper Silesia), 12 Jul 2017, No. 07/17/2a, on *Ulmus glabra* ‘Pendula’, coll. Kanturski (UŚ); two alate viviparous females, SWEDEN: Lund, 03 Sep 1978, No. R.3963, on *Ulmus glabra*, coll. R. Danielsson (UŚ); six alate viviparous females, UNITED KINGDOM: ex culture of G. Hopkins, 04 Oct 1995, No. RLB 4337, on *Ulmus* sp., coll. R. Blackman (BMNH).

### Key to the species of the genus *Tinocallis* in Europe

**Table:** 

1	Live specimens: head and thorax yellow or brown, abdomen yellow with or without brown patches. Mounted specimens: head and pronotum without projections (Fig. 4b). Mesonotum without projections. PT shorter than 0.50 × BASE	**2**
–	Live specimens: head and thorax from yellow to dark. If brown to dark, then abdomen only with small circular sclerites and scleroites. Mounted specimens: pronotum with one or two pairs of finger-like projections (Figs 4a, c). Mesonotum with one pair of projections. PT as long as or longer than 0.50 × BASE	**3**
2	Live specimens: head and thorax brown, abdomen yellow with extensive brown patches (Fig. 1b). Mounted specimens: forewings pigmented, hind femora brown, abdomen with extensive, dark sclerotisation (Fig. 2b)	***T. platani***
–	Live specimens: pale yellow without any patches. Mounted specimens: forewings not pigmented, hind femora pale, abdomen without dark sclerotisation	***T. zelkowae***
3	Live specimens: head and thorax black, abdomen yellow without dark sclerites (Fig. 1a, c). Pronotum with one pair of projections on distal part. Forewings pigmented. Hind legs with brown distal part of femora and proximal part of tibiae (Fig. 7a)	***T. takachihoensis***
–	Live specimens: head and thorax greenish or yellow to brown, abdomen with small to large circular sclerites or marginal projections. Mounted specimens: pronotum with two pairs of projections on proximal and distal part. Forewings pigmented or not pigmented. Hind legs with pale femora only with a brown spot or band on the distal part and pale tibiae (Fig. 7c, d)	**4**
4	Live specimens: greenish or bluish-green. Mounted specimens: Head with all projections prominent, the distal pair almost as long as the projections on the pronotum. Marginal projections on ABD II-IV almost as long as the spinal projections	***T. ulmiparvifoliae***
–	Live specimens: yellow to brown. Mounted specimens: Head with small projections, the distal pair clearly shorter than projections on pronotum. Marginal projections on ABD II-IV clearly shorter than spinal projections	**5**
5	Live specimens: head and thorax brown, abdomen with clearly visible sclerites with projections or tubercles, wings clearly pigmented (Fig. 1e). Mounted specimens: ANT III secondary rhinaria transverse oval (Fig. 3c). Wings clearly pigmented. Abdomen with small, sclerotised, spinal projections on ABD IV (Fig. 8c)	***T. saltans***
–	Live specimens: head and thorax yellow or thorax brown with poorly visible sclerites, wings not pigmented. Mounted specimens: ANT III secondary rhinaria slit-like (Fig. 3d). Wings very poorly pigmented. Abdomen without small sclerotised spinal projections on ABD IV (with only two sclerites) (Fig. 8d)	***T. nevskyi***

### Distribution comments

Of the approximately 18 valid *Tinocallis* species, six are known from Europe (Blackman and Eastop 2017). Although Nieto Nafría et al. (2013) also include *Sarucallis kahawaluokalani* (Kirkaldy, 1906) as a representative of *Tinocallis* (in the subgenus Sarucallis), we follow the full generic status of *Sarucallis* (Quednau 2003). Two of the *Tinocallis* species known from Europe, *T. ulmiparvifoliae* and *T. zelkowae*, are only known from south-western Europe and on bonsai trees imported into the United Kingdom, while the remaining taxa have been reported to occur more widely, especially in central and northern Europe. From the four species presented here, *T. platani* is characterised as being widely distributed, *T. nevskyi* in the central, western, and northern parts of the continent, while *T. saltans* is recorded mostly in the central and eastern parts of Europe, but also from Italy and Spain (Nieto Nafría and Mier Durante 1998). Earliest European records of *T. takachihoensis* were from southern France and Italy, with more recent records from Greece, Germany, Malta, and the Netherlands. Thus, it seems from the dispersion routes of this species in Europe, that for more than a decade it has preferred regions with milder climate (the Mediterranean Basin and the western coast of Europe). Its sexual generation is as yet only known from Malta (Patti and Barbagallo 1997). The recent finding in Central Europe indicates the possibility of either the overwintering of this species in the climatic conditions of this part of Europe or rapid early-summer migrations from the territory that is already occupied (Piron 2013). Despite some milder winters, which have occurred in Central Europe recently and have influenced aphid biology (Depa et al. 2015), the period of aphid collection in this case (June–August) indicates the second possibility. The species, however, requires observation as it may become a pest on ornamental plants or it may adapt to more severe climatic conditions.

## Supplementary Material

XML Treatment for Tinocallis

XML Treatment for Tinocallis (Sappocallis) takachihoensis

XML Treatment for Tinocallis (Eotinocallis) platani

XML Treatment for Tinocallis (Sappocallis) saltans

XML Treatment for Tinocallis (Sappocallis) nevskyi
